# Survey of physician knowledge regarding antiretroviral medications in hospitalized HIV-infected patients

**DOI:** 10.1186/1758-2652-12-1

**Published:** 2009-02-02

**Authors:** Saarah Arshad, Michael Rothberg, Darius A Rastegar, Linda M Spooner, Daniel Skiest

**Affiliations:** 1 Infectious Disease Division, Baystate Medical Center-Tufts University School of Medicine, Springfield, Massachusetts, USA; 2 General Medicine and Geriatrics, Baystate Medical Center-Tufts University School of Medicine, Springfield, Massachusetts, USA; 3 Johns Hopkins Bayview Medical Center, Baltimore, Maryland, USA; 4 Massachusetts College of Pharmacy and Health Sciences-School of Pharmacy-Worcester/Manchester, Worcester, Massachusetts, USA

## Abstract

**Background:**

Antiretroviral prescribing errors are common among hospitalized patients. Inadequate medical knowledge is likely one of the factors leading to these errors. Our objective was to determine the proportion of hospital physicians with knowledge gaps about prescribing antiretroviral medications for hospitalized HIV-infected patients and to correlate knowledge with length and type of medical training and experience.

**Methods:**

We conducted an electronic survey comprising of ten clinical scenarios based on antiretroviral-prescribing errors seen at two community teaching hospitals. It also contained demographic questions regarding length and type of medical training and antiretroviral prescribing experience. Three hundred and forty three physicians at both hospitals were asked to anonymously complete the survey between February 2007 and April 2007.

**Results:**

One hundred and fifty-seven physicians (46%) completed at least one question. The mean percentage of correct responses was 33% for resident physicians, 37% for attending physicians, and 93% for Infectious Diseases or HIV (ID/HIV) specialist physicians. Higher scores were independently associated with ID/HIV specialty, number of outpatients seen per month and physician reported comfort level in managing HIV patients (P < .001).

**Conclusion:**

Non-ID/HIV physicians had uniformly poor knowledge of common antiretroviral medication regimens. Involvement of ID/HIV specialists in the prescribing of antiretrovirals in hospitalized patients might mitigate prescribing errors stemming from knowledge deficits.

## Introduction

Medication errors are common, harming at least 1.5 million people in the United States every year and costing billions of dollars annually [1]. These errors can occur at levels of prescribing, dispensing and/or administration. Many factors have been associated with prescribing errors, including: inadequate knowledge of the prescriber; inadequate access to information; sound-alike medication names; incorrect dosage or dose frequency; inaccurate adjustment for hepatic or renal impairment; complicated regimens; and incorrect reporting by the patient [2-5]. Medication errors frequently occur at the time of hospital admission, mostly due to omission of regularly used medication [6]. Errors in conversion of outpatient HIV medications to the hospital's formulary-equivalent drugs have been shown to be associated with moderate to severe discomfort or clinical deterioration of HIV patients [7].

The use of combination antiretroviral therapy (ART) has led to major improvements in the management of HIV/AIDS in the developed world and increasingly in the developing world. A minimum of three agents are typically utilized in antiretroviral regimens. Even with the use of external resources, it is difficult at times to precisely dose antiretroviral medication due to complex drug-drug interactions and adverse effects.

Successful management of HIV requires close adherence to recommended ART prescribing guidelines. Antiretroviral prescribing errors may result in actual or potential patient harm, including treatment failure, emergence of resistance and toxicity [3,8,9]. Previous studies showed ART prescribing errors in HIV-infected hospitalized patients in as many as 26% of admissions [3-5]. In a more recent study, at least one error was seen in the initial medication regimen of 72% of HIV-infected patient admissions [7].

With the advent of highly active antiretroviral therapy, HIV has become a chronic disease, primarily managed in the outpatient setting by HIV specialists. As a result, non-HIV specialists working in hospitals may have little occasion to initiate ART and may not be familiar with increasingly complex regimens. In order to discern whether lack of knowledge and experience might account for antiretroviral medication prescribing errors, we conducted a study to assess the knowledge of physicians prescribing antiretroviral medications in hospitalized patients. We hypothesized that general internists would have limited knowledge of antiretroviral regimens, whereas infectious disease physicians and HIV-experienced internists would have adequate knowledge of these medications.

## Methods

We conducted an anonymous survey (see appendix 1) at two community teaching hospitals: Baystate Medical Center (BMC), a 653-bed tertiary care hospital in Springfield, Massachusetts and Johns Hopkins Bayview Medical Center (JHBMC), a 354-bed hospital in Baltimore, Maryland. Both hospitals use a computerized provider order entry system. Both hospitals have active residencies in internal medicine, pediatrics and family practice, as well as fellowship programmes in infectious diseases. The study was approved by the Institutional Review Board at each hospital. Two other hospitals originally participating in the study were excluded due to very low response rate to the survey: one prior to IRB approval and the other prior to data analysis. The data from these two institutions were not reviewed prior to their exclusion.

The survey was sent to all residents, fellows and attending physicians in the divisions of General Internal Medicine, Medicine/Pediatrics, Family Practice, Critical Care and Infectious Diseases at both hospitals. The survey was sent as a hyperlink in an emailed invitation letter to a total of 343 physicians at both hospitals (210 at BMC and 133 at JHBMC) between February 2007 and April 2007. Two to three reminder letters were emailed to the physicians.

The survey, created by the authors using SurveyMonkey.com (an online survey tool, based in Portland, Oregon), was divided into two sections. One contained basic demographic questions, including: level of training; current position; specialization in Infectious Diseases (ID) or HIV; number of years elapsed since residency; number of HIV inpatients seen per month; number of HIV outpatients seen per month; percentage of time spent seeing inpatients per year; number of changes made in antiretroviral medications in the previous one month; and the level of comfort in managing HIV patients (ranging between 1 and 5 with 1 = not comfortable and 5 = extremely comfortable).

The second section included 10 multiple choice questions derived from commonly encountered antiretroviral medication prescribing errors observed by HIV clinicians and pharmacists at the two hospitals [3,4]. The questions assessed knowledge of ART dosing (one question), frequency (three questions), renal dosage adjustment (one question), drug interactions (four questions) and omission of an antiretroviral medication (one question). The questions were reviewed by several ID and non-ID physicians and pharmacists for clarity, interpretability and accuracy.

The Department of Health and Human Services' Guidelines for the Use of Antiretroviral Agents in HIV-1-Infected Adults and Adolescents were used as the primary reference to determine if the regimen was correct [10]. Each antiretroviral medication-related question was scored with one point if answered correctly and zero points if answered incorrectly. At the beginning of the survey, physicians were informed that it was anonymous and were instructed not to use external resources to answer questions. The survey design did not allow skipping of questions, and respondents could exit the survey at any time.

### Statistical analysis

Participants were divided into three groups for comparison: residents; attending physicians (included non-ID/HIV specialist attending physicians and non-ID Fellows); and ID/HIV specialist physicians (included ID fellows, ID attending physicians and non-ID attendings who identified themselves as HIV specialists). Mean knowledge scores were used for different categories of discrete factors and Pearson correlation coefficients for continuous factors. Factors in mean knowledge score were analyzed using Analysis of Covariance [11]. Grouping factors included: designation as an ID/HIV specialist; physician type (resident, attending or specialist); number of inpatients and outpatients seen per month; and percent of inpatients seen per year. Years since completion of residency, comfort level and number of changes made in the past month were included as covariates.

## Results

**Figure 1:**
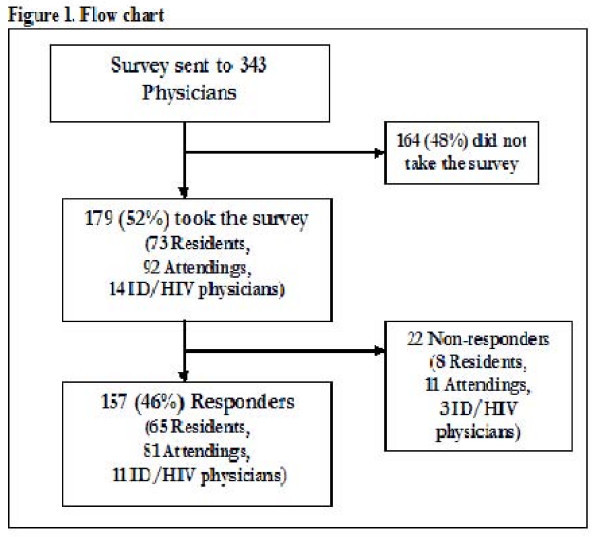
**Flow chart**.

Of 343 physicians who received the email request, 179 (52%) completed at least the demographic section of the survey (98 from BMC and 81 from JHBMC). Physicians who answered at least one antiretroviral medication-related question were similar demographically to those who did not answer any questions. Thus, the 22 physicians who did not answer any of the antiretroviral medication-related questions were excluded from the analysis (figure 1).

Respondents included 65 residents, 81 attending physicians and 11 ID/HIV physicians. Of these respondents 142 (90%) answered all 10 questions. Nine answered one to five questions and six answered six to nine questions. The respondents who left the survey before completing all the antiretroviral-related questions were evaluated in two ways: unanswered questions received a score of zero; and unanswered questions were not counted. Since both methods yielded similar results, we report only the percentage of answered questions.

Basic demographics of the physicians surveyed are shown in Table 1. A majority had completed residency training in the past 10 years. Less than 25% of residents and non-ID/HIV physicians reported having a comfort level of ≥ 3 for managing HIV-infected patients, as compared to 100% of ID/HIV specialists (*P *< 0.05). Half of all physicians surveyed saw one to five hospitalized HIV patients per month, whereas 36% of ID/HIV physicians saw ≥ 10 hospitalized HIV patients per month. Less than 10% of residents and non-ID/HIV attending physicians reported changing or starting any antiretroviral medications in the past month compared to 100% of ID/HIV specialists (*P *< 0.05).

**Table 1:** Demographic characteristics of survey respondents

	Physician type		
	
Characteristic	Resident n = 65 (%)	Non ID/HIV attending physician n = 81 (%)	ID/HIV physician n = 11 (%)
**No. of years since residency completed**			
≤ 5		30 (37)	3 (30)
6–10		15 (19)	2 (20)
11–20		23 (28)	5 (50)
> 20		13 (16)	0
			
**Residency level (post-graduate year)**			
PGY1	21 (32)		
PGY2	18 (28)		
PGY3	20 (31)		
PGY4	6 (9)		1 (100)
			
**No. of HIV inpatients/month**			
0	2 (3)	24 (30)	
1–5	51 (78)	51 (63)	6 (55)
6–10	12 (18)	4 (5)	1 (9)
11–20		2 (2)	2 (18)
> 20			2 (18)
			
**No. of HIV outpatients/month**			
0	30 (46)	59 (73)	
1–5	31 (48)	16 (20)	2 (18)
6–10	3 (5)	4 (5)	
11–20	1 (2)	1 (1)	3 (27)
> 20		1 (1)	6 (55)
			
**% inpatients/yr**			
1–25%	9 (14)	49 (60)	7 (64)
26–50%	17 (26)	7 (9)	1 (9)
51–75%	28 (43)	6 (7)	1 (9)
76–100%	11 (17)	19 (23)	2 (18)
			
**HIV related "comfort level"**			
1	18 (28)	23 (28)	
2	38 (58)	40 (49)	
3	8 (12)	15 (19)	4 (36)
4	1 (2)	3 (4)	2 (18)
5			5 (45)
			
**No. of changes/start of HIV medications in past month**			
0	59 (91)	73 (90)	0
1–2	4 (6)	6 (7)	2 (18)
≥ 3	2 (3)	2 (2)	9 (82)

**Figure 2:**
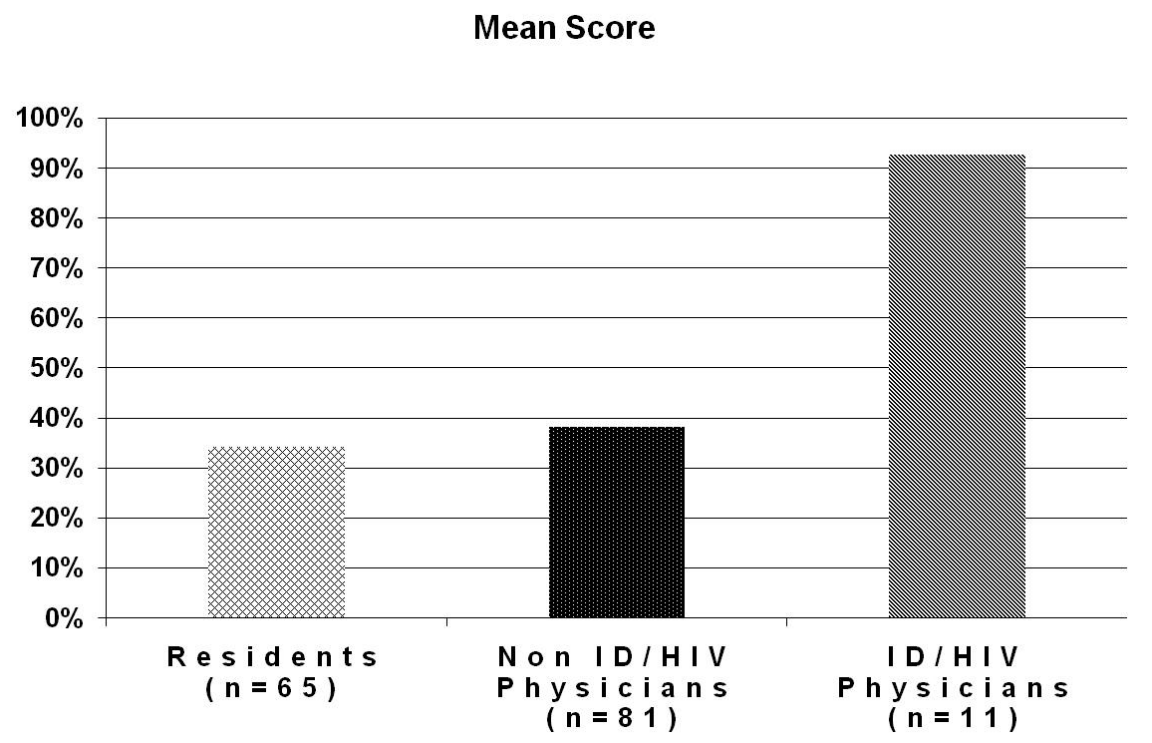
**Mean score (percent of survey questions answered correctly) for residents, attendings and ID/HIV physicians**.

**Figure 3:**
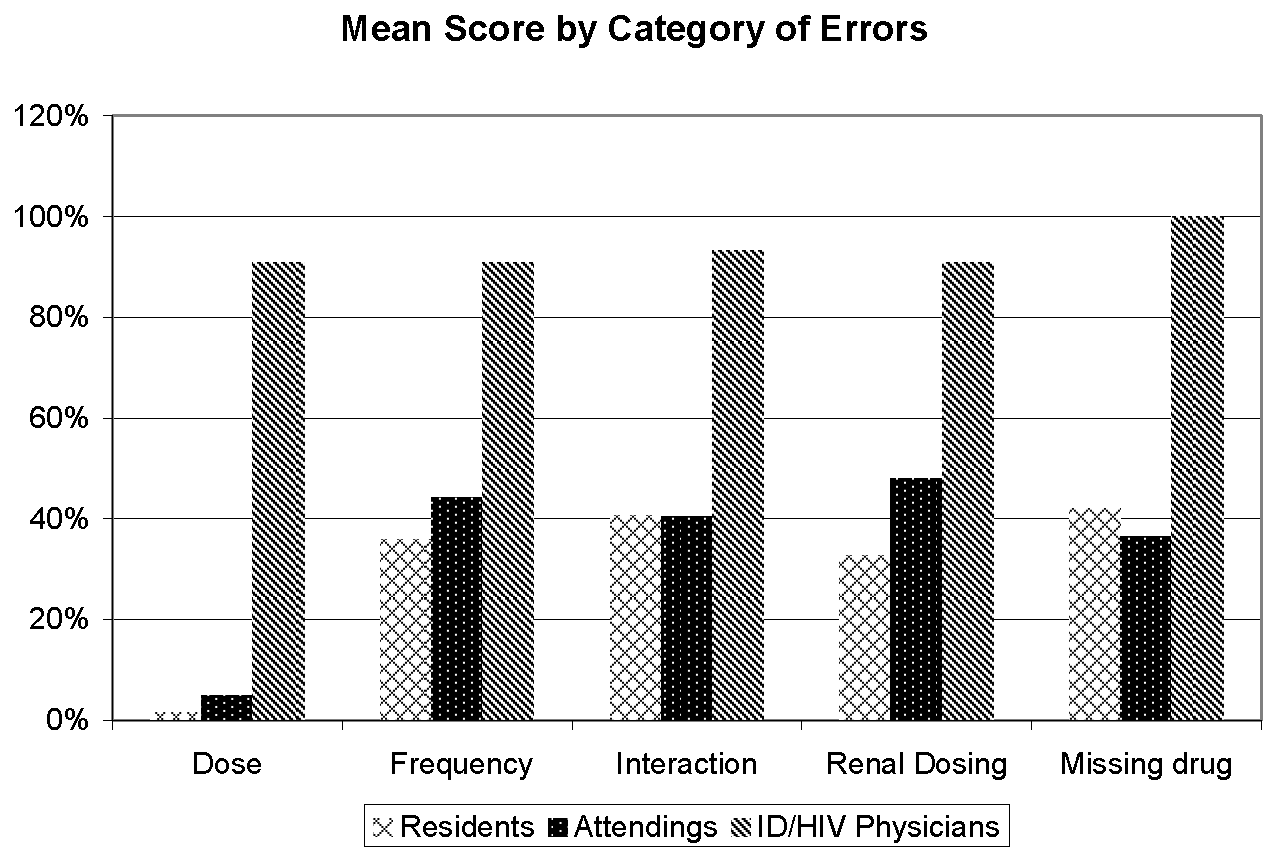
**Mean score (percent of survey questions answered correctly) for residents, attendings and ID/HIV physicians based on the category of errors**.

The median score for the antiretroviral medication-related questions answered correctly was 30% (range = 0–80%) for both residents and attending physicians compared to 90% (range = 80–100%) for ID/HIV specialist physicians (Figure 2). Scores were similar across all categories of errors, except for dosing (Figure 3). Non-ID/HIV physicians as a group scored less than 6% on the dosing question (*P *< 0.05). No difference was found when scores from BMC and JHBMC were compared (*P *> 0.9).

Table 2 shows the univariate association of each factor with knowledge scores. The results of analysis of covariance showed that three factors were significantly related to HIV knowledge: designation as an ID/HIV specialist (*P *< 0.001); number of outpatients seen per month (*P *< 0.001); and comfort level in managing HIV patients (*P *< 0.001). These variables combined explained 50% of the variance in knowledge scores (adjusted r^2 ^= 0.50). The mean score for ID/HIV specialists was 93%, compared to only 35% for non-ID/HIV specialists (including residents and attendings).

**Table 2:** Factors associated with correct survey answers: univariate analysis

Demographics	Mean Score	*P *value*
**Specialty**		
ID/HIV physicians (n = 11)	93%	< 0.05
Non-ID/HIV physicians (n = 146)	35%	
**No. of years since residency**		
≤ 10	44%	0.79
> 10	42%	
**No. of HIV inpatients/month**		
≤ 5	37%	< 0.05
> 5	55%	
**No. of HIV outpatients/month**		
≤ 5	35%	< 0.05
> 5	72%	
**Comfort level**		
1–2	33%	< 0.05
> 2	58%	
**Survey site**		
BMC (n = 82)	39%	> 0.9
JHMBC (n = 75)	39%	

* calculated by *t*-test

There was a trend for scores to increase with increasing number of inpatient visits but it did not reach statistical significance (*P *= 0.10). There was a positive correlation between number of changes made in medications in the past month and knowledge scores (r = 0.55). Differences by training level (ID/HIV specialist, attending or resident) were accounted for by ID/HIV specialty. There was little correlation between the number of years since residency and test performance. (r = 0.128).

## Discussion

HIV/AIDS is now considered a chronic disease, which can be successfully managed with appropriate use of antiretroviral medications in most individuals. Adherence to the antiretroviral regimen is a major factor in the success of treatment [8,9]. We found that among non-HIV specialists who care for hospitalized HIV-infected patients, knowledge of antiretroviral regimens was poor. Furthermore, knowledge of attending physicians was no better than that of residents. Few respondents were able to answer more than 40% of questions correctly, and knowledge of dosing was particularly poor. In contrast, ID/HIV specialists had excellent knowledge, as indicated by better scores, and always identified incomplete drug regimens.

Prior studies have demonstrated that levels of adherence of 95% or greater are required to prevent regimen failure due to the development of viral resistance [9]. Historically, efforts have focused on ART adherence in the outpatient setting. However, it is also important to ensure that correct ART medications are dispensed during hospitalization and particularly at discharge, since patients may continue incorrect regimens without the knowledge of their HIV providers.

Without sufficient knowledge of rapidly changing antiviral regimens, hospital physicians, who rarely initiate or change antiviral therapy, must rely in most cases on patients' recollection of their regimens, which may not be accurate. Previous studies done at our hospitals found that 21% to 26% of HIV patients experienced ART prescribing errors during the hospitalization [3,4].

Because of the large number of prescribers, each with a small number of patients, as well as frequently changing regimens, educational interventions aimed at hospital physicians are not practical. One potential solution is mandatory consultation with an ID/HIV specialist. Previous studies have shown that HIV-specific knowledge is strongly associated with HIV caseload [12], and that non-infectious disease HIV specialists perform as well as Infectious Disease physicians [12-15]. Both of these findings were also observed in our study.

Similarly, we found that ID/HIV physicians made fewer errors than non-ID/HIV physicians while managing hypothetical hospitalized HIV patients, which to our knowledge, has not been previously demonstrated. Partly in response to our experience with HIV prescribing errors, we have implemented mandatory ID consultation for all HIV-infected inpatients.

Another intervention to decrease ART errors is the use of a standardized antiretroviral order set. We have recently implemented such an order set, in which standardized doses and ART regimens are suggested during the ordering process. Unusual doses or ART combinations have to be ordered separately in an attempt to minimize errors. This system appears to have resulted in fewer errors (unpublished data).

Clinical pharmacists trained in HIV may be able to prevent and mitigate ART errors by "catching" them early, hopefully before any harm is done. HIV clinical pharmacists may also be helpful educational resources for residents and attending physicians while making decisions about ART medications. At our hospital, an HIV clinical pharmacist regularly reviews the antiretroviral medication regimen for HIV-infected inpatients. A study done at our hospital showed a reduction in duration of antiretroviral-related errors in hospitalized patients with the interventions of a clinical pharmacist [4].

Our study highlights the difficulties of medication reconciliation when the patient is not certain of their medical regimen. With the advent of hospitalists, who now care for the majority of inpatients at most US hospitals, there is a danger that inappropriate regimens will have started in the hospital and continued at discharge simply because the prescribers are unfamiliar with outpatient medications.

This study had several limitations. First, the small number of participating ID/HIV physicians may not be representative of all HIV specialists. However, there was little variation in the scores of the HIV specialists, and those few specialists tasked with HIV consults at other institutions would likely have similar expertise. What is more surprising is how uniformly low the scores are of the non-HIV specialists, who do the bulk of HIV prescribing in the hospital.

Second, no information was available for the 164 (48%) non-responding physicians. It is likely that those who did not respond would have scored the same or worse than those who did respond, accentuating the difference seen between specialists and non-specialists.

Third, our study was limited to two academic hospitals. It is possible that our results be not be applicable to other clinical settings. However, our two hospitals were of different sizes, and in different states; yet the knowledge levels based on the survey responses in both were remarkably similar.

Fourth, our survey has not been validated. Thus, we can not definitively conclude that clinicians caring for HIV-infected inpatients should possess this knowledge. However, the questions were based on previously published common antiretroviral prescribing errors made by clinicians (3, 4). We think it is important for clinicians to recognize these common scenarios.

Finally, physicians were asked not to use external resources to search for answers to the questions. In practice, such resources are available, which may positively impact on appropriate ART prescribing, and allowance of the use of such resources may result in better antiretroviral prescribing knowledge. Indeed, previous studies in both hospitals found HIV prescribing errors in one quarter of all HIV admissions.

Antiretroviral prescribing errors seen in hospitalized HIV patients, including incorrect dosage and incomplete regimens, are common and could lead to antiretroviral resistance. Based on our study, knowledge deficits among non-HIV specialists may potentially contribute to these errors. Because educational interventions alone may not be sufficient, consideration should be given to other interventions, such as mandatory ID/HIV consultation, standardized orders sets, and review by an HIV clinical pharmacist, in order to decrease the frequency of such errors. Improvement in information systems that facilitate the continuation of medications from one setting to another may also help prevent some of these errors. Further studies are warranted to look at the actual or potential harm resulting from knowledge deficits and analyze the potential benefits of these interventions.

## Competing interests

The authors declare that they have no competing interests.

## Authors' contributions

SA – conceived of the project, collected data, analyzed data, wrote manuscript; MR – conceived of the project, analyzed data, edited manuscript; DAR – collected data, edited manuscript; LMS – collected data, edited manuscript; DS – conceived of the project, analyzed data, co-wrote manuscript.

## Appendix

### Appendix 1 – HIV Survey

#### Demographics

1) What is your specialty?

         a. Hospitalist

         b. Internal Medicine

         c. Family Practice

         d. Infectious Diseases

         e. Med/Peds

         f. Other (please specify)

2) What is your current position?

         a. Resident

         b. ID fellow

         c. Attending physician

3) If you are a resident?

      A. Year of residency?

         a. R1

         b. R2

         c. R3

         d. R4

3) If you are an attending physician?

      a) Do you consider yourself an HIV specialist?

         a. Yes

         b. No

      b) Number of years since you completed your residency?

         a. < 3 years

         b. 3–5 years

         c. 6–10 years

         d. 11–20 years

         e. > 20 years

4) How many HIV+ inpatients (new and existing) do you see per month on average?

         a. Zero

         b. 1–5

         c. 6–10

         d. 11–20

         e. > 20

5) How many HIV+ outpatients (new and existing) do you see per month on average?

         a. Zero

         b. 1–5

         c. 6–10

         d. 11–20

         e. > 20

6) What percentage of time do you spend seeing inpatients per year?

         a. 1–25%

         b. 26–50%

         c. 51–75%

         d. 76–100%

7) How comfortable do you feel managing HIV patients, on a scale of 1 to 5, with 1 being not at all comfortable and 5 being extremely comfortable?

         Scale 1–2–3–4–5

8) In the past ONE month, for how many patients have you initiated or changed antiretroviral medications? ↑

Please choose the singlebest answer:

#### Questions

Q1. 53 yo HIV+ man is admitted to the hospital for cellulitis of the leg. He states he takes Truvada (tenofovir + emtricitabine) once a day and Sustiva (efavirenz) 200 mg at bedtime. During the admission it is discovered that he has hepatitis C with moderate cirrhosis. You should:

      a.   Continue with current regimen

      b.   Change Sustiva to 100 mg at bedtime

      c.   Change Sustiva to 600 mg at bedtime

      d.   Hold Sustiva

Q2. 41 yo HIV+ man receiving Combivir (zidovudine + lamivudine) and Viramune (nevirapine) for the past many years is admitted to the hospital for pneumonia. He has normal creatinine clearance. The admitting doctor ordered Combivir 300/150 mg once a day and Viramune 200 mg twice a day. You should:

      a.   Continue with current regimen

      b.   Change Viramune to once a day

      c.   Change Combivir to twice a day

      d.   Change Combivir to three times a day

Q3. 35 yo HIV+ man presents to ED with a list of herbal remedies he takes for a variety of medical conditions. His current medications include: Truvada (tenofovir + emtricitabine) and Kaletra (lopinavir + ritonavir). Which of the following herbal remedies is contraindicated for use with his current antiretroviral regimen?

      a.   garlic

      b.   zinc

      c.   gingko

      d.   St. John's wort

      e.   saw palmetto

Q4. 49 yo HIV+ treatment experienced man reports taking Combivir (zidovudine + lamivudine) 300/150 mg once a day, Lexiva (fosamprenavir) 700 mg once a day and Norvir (ritonavir) 200 mg once a day at home. He is admitted to the hospital for elective cholecystectomy. He has normal creatinine clearance. You should:

      a.   Continue with current regimen

      b.   Hold HIV medications until after surgery

      c.   Change regimen to Combivir twice a day, Lexiva 700 mg twice a day and Norvir 100 mg twice a day

      d.   Change regimen to Combivir twice a day and Lexiva 700 mg twice a day

Q5. 55 yo man is admitted to the ICU with cardiogenic shock due to myocardial infarction and acute renal failure. His creatinine clearance is less than 10 ml/minute. His antiretroviral therapy includes Ziagen (abacavir) 300 mg twice a day, Epivir (lamivudine) 150 mg twice a day and Sustiva (efavirenz) 600 mg at bedtime. You should:

      a.   Continue with current regimen

      b.   Change Ziagen to 150 mg daily

      c.   Change Epivir to 50 mg daily

      d.   Change Sustiva to 300 mg daily

Q6. 35 yo HIV+ man former injecting drug user has been receiving Methadone 50 mg PO daily for past six months. He is admitted to the hospital at 3 am on Saturday for anxiety, vomiting, tachycardia and hypertension. He started antiretroviral therapy one week ago with Combivir (zidovudine + lamivudine) and Sustiva (efavirenz). You are unable to reach his ID provider over the weekend. You should recommend:

      a.   Continuing with current regimen

      b.   Increasing dose of Methadone

      c.   Holding Sustiva

      d.   Holding Combivir

Q7. 61 yo man with h/o hyperlipidemia, diabetes mellitus and HIV is admitted to the hospital for uncontrolled diabetes. He takes Kaletra (lopinavir + ritonavir) and Epzicom (abacavir + lamivudine) for HIV and insulin for diabetes. All medications are continued. His CD4 count has been stable around 450 cells/mm with HIV viral load < 50 copies/ml. He is started on atorvastatin 20 mg once a day for hyperlipidemia in the hospital. You should:

      a.   Continue with current regimen

      b.   Change atorvastatin to simvastatin 40 mg

      c.   Change atorvastatin to lovastatin 40 mg

      d.   Change Kaletra to ritonavir alone

Q8. 39 yo HIV+ man is admitted to the hospital for an ankle fracture. He recalls taking Combivir (zidovudine + lamivudine) 300/150 mg one pill twice a day and Kaletra (lopinavir + ritonavir) 200/50 mg two pills twice a day at home. He has normal creatinine clearance. You should:

      a.   Continue with current regimen

      b.   Change to Combivir one pill once a day and Kaletra one pill once a day

      c.   Change to Combivir one pill twice a day and Kaletra one pill twice a day

      d.   Change to Combivir one pill twice a day and Kaletra three pills twice a day

Q9. 57 yo HIV+ man is admitted to the hospital for abdominal pain. He has been taking antiretroviral therapy for one year. Two weeks ago his CD4 count was stable at 350 cells/mm^3^. On admission the resident orders Reyataz (atazanavir) 400 mg once a day and Truvada (tenofovir + emtricitabine) once a day. You should:

      a.   Continue with current regimen

      b.   Change Reyataz to 300 mg daily

      c.   Add Norvir (ritonavir) 100 mg daily

      d.   Change Reyataz to 300 mg daily and add Norvir (ritonavir) 100 mg daily

Q10. 53 yo HIV + woman admitted for elective knee replacement. She states she takes one pill for HIV at bedtime and her HIV viral load has been < 50 copies for several months. The medication causes bizarre dreams but she takes it regularly. The physician's assistant orders Sustiva (efavirenz) on admission. You are called to review her medications on Friday evening. You should:

      a.   Continue Sustiva

      b.   Change to Retrovir (zidovudine) 300 mg at bedtime

      c.   Change to Atripla (efavirenz + tenofovir + emtricitabine) one pill at bedtime

      d.   Hold Sustiva until after surgery
